# Research on Cutting Layer Characteristics of Superalloy under High-Pressure Cooling

**DOI:** 10.3390/ma16113931

**Published:** 2023-05-24

**Authors:** Lubin Li, Shuning Chen, Tiankang Li, Mingyang Wu

**Affiliations:** 1 School of Mechanical Engineering, Heilongjiang University of Science and Technology, Harbin 150022, China; qishilifei@163.com (L.L.); litiankang0724@gmail.com (T.L.); 2 Key Laboratory of National and Local United Engineering for High-Efficiency Cutting and Tools, Harbin University of Science and Technology, Harbin 150080, China; 13936161878@139.com

**Keywords:** superalloy, high-pressure cooling, PCBN tools, characteristics of cutting layer

## Abstract

Superalloys are widely used in the aerospace field and are a typical difficult-to-cut material. When the PCBN tool is used to cut superalloys, there will be problems such as a large cutting force, a high cutting temperature, and gradual tool wear. High-pressure cooling technology can effectively solve these problems. Therefore, this paper carried out an experimental study of a PCBN tool cutting superalloys under high-pressure cooling and analyzed the influence of high-pressure coolant on the characteristics of the cutting layer. The results show that the main cutting force can be reduced by 19~45% and 11~39% when cutting superalloys under high-pressure cooling compared with dry cutting and atmospheric pressure cutting, respectively, in the range of test parameters. The surface roughness of the machined workpiece is less affected by the high-pressure coolant, but the high-pressure coolant can help reduce the surface residual stress. The high-pressure coolant can effectively improve the chip’s breaking ability. In order to ensure the service life of PCBN tools, when cutting superalloys under high-pressure cooling the coolant pressure should not be too high, and 50 bar is more appropriate. This provides a certain technical basis for the efficient cutting of superalloys under high-pressure cooling conditions.

## 1. Introduction

With the rapid development of the aviation industry, the proportion of superalloys being used is increasing, especially nickel-based superalloys. In high-temperature environments, they maintain good tensile strength, yield strength, fatigue resistance, high oxidation resistance, good corrosion resistance, and low creep. Therefore, they are widely used in aerospace, petrochemical, nuclear power, shipbuilding, and other industries [1]. However, the materials of superalloys have low thermal conductivity as well as high microhardness and shear stress resistance, which increase their cutting force and temperature during the cutting process [2], resulting in tool wear, a decrease in the surface quality of the workpiece, and other phenomena leading to difficult cutting. Nowadays, the materials used in cutting nickel-based superalloys are generally polycrystalline cubic boron nitride (PCBN), cemented carbide, and ceramics. When it comes to cutting hard-to-cut materials, PCBN tools have some advantages, so they are widely used [3].

Li et al. [4] carried out dry cutting experiments of superalloys. The results showed that the cutting temperature of the workpiece during dry cutting was high and the tool wear was serious. Moreover, PCBN tools generally have flat rake face, no-chip breaking grooves, and easily lead to chip-breaking difficulties, which will affect the machining accuracy and efficiency of superalloys. Therefore, it is of great significance to explore new and efficient heat transfer technologies and improve the heat transfer efficiency of coolants in the cutting zone to achieve efficient processing and high surface quality of nickel-based superalloys. Many scholars worldwide have carried out research on cutting cooling technology, such as micro lubrication [5], low-temperature cooling [6], heat pipe cooling [7], liquid nitrogen cooling [8], spray cooling [9], water vapor cooling [10], and other methods. These cooling technologies can lead to better cooling and lubrication effects. However, these cooling cutting techniques have less obvious effects on chip breaking and are technically complex and costly. In order to further improve cooling and lubrication technology, high-pressure cooling-assisted cutting has become an effective method [11]. When using high-pressure coolants for auxiliary cutting, the coolant is sprayed out of a small-diameter hole, which means the cutting area can be more easily poured by the coolant, reducing the cutting temperature, and thus improving the tool life [12]. Meanwhile, the impact characteristic of the high-pressure coolant is conducive to chip breaking [13]. Therefore, this paper adopts a high-pressure cooling lubrication method for cutting GH4169. Wu [14] and Li [15] et al. carried out experimental research on cutting the superalloy GH4169 under high-pressure cooling. The research results confirmed that the high-pressure coolant can effectively reduce tool wear, reduce the curling radius of the chip, and improve the chip’s breaking ability. Alagan et al. [16] studied the effect of cutting Alloy 718 tool micro-texture on cutting performance under high-pressure cooling. The results showed that the use of high-pressure cooling assistance and micro-texture can help improve the service life of the tool. Fang et al. [17] studied the effect of a high-pressure coolant channel on the cutting performance of turning Inconel 718 based on a novel internally cooled insert. The results showed that using a novel high-pressure internal cooling insert can improve the surface quality of the workpiece. Liu et al. [18] conducted an experimental study on interrupted cutting of Inconel 718 with AlTiSiN-coated cemented carbide tools under high-pressure cooling. The results indicated that high-pressure cooling effectively reduced the temperature of the cutting zone, reduced the diffusion and associated flaking of the tool substrate material, and effectively suppressed the wear of interrupted cutting tools. Wu et al. [19] found, through experimental research on PCBN cutting tools cutting nickel-based superalloy under high-pressure cooling, that the cutting bending moment increased, the chip strain increased, and the chip strain quickly reached the material limit strain and broke during high-pressure cooling processing. Alagan et al. [20] conducted turning experiments on Alloy 718 under high-pressure cooling. The results showed that a coolant pressure of 16 MPa was more effective in reducing tool wear than a coolant pressure of 8 MPa. Nasr et al. [21] conducted cutting experiments on high-pressure cooling-assisted machining of Inconel 625. The results showed that under optimal coolant pressure, adhesion wear can be minimized. Díaz-Álvarez et al. [22] studied the finishing turning of Haynes 282 under high-pressure cooling and found that under high-pressure cooling, when the cutting speed is 50 m/min and the feed is 0.1 mm/r, the workpiece can obtain the best surface finishes. Wang et al. [23] found through experimental research on cutting the superalloy GH4169 with PCBN tools under high-pressure cooling that high-pressure cooling can reduce chip length, curl radius, and chip width. Li et al. [24] conducted cutting experiments on high-temperature alloy GH4169 using high-pressure cooling technology and studied the effects of different cutting parameters and cooling pressure on surface roughness and tool life. The results showed that the feed rate had the greatest influence on the surface roughness, and the cutting speed had the greatest influence on the tool life. Sørby et al. [25] used ceramic cutting tools to cut Inconel 625 under high-pressure cooling. The results showed that high-pressure cooling was helpful for chip breaking. Wu et al. [26] established a tool stress model under high-pressure cooling for simulation and conducted experiments to verify it. The results showed that high-pressure cooling can effectively reduce tool stress, and the tool stress is the smallest when the coolant pressure is 5 MPa. Alagan et al. [27] found through experimental research on cutting Alloy 718 under high-pressure cooling that adjusting the coolant pressure towards the tool flank to maintain it above the vapor pressure of the coolant for the corresponding cutting temperature can avoid coolant boiling and cavitation wear on the tool flank. Wu et al. [28] studied the effect of chip plastic side flow on the rake face of a PCBN tool under high-pressure cooling. The results showed that high-pressure cooling can reduce the wear of the tool rake face caused by chip plastic side flow. Wei et al. [29] found that by studying the influence of tool wear on the microstructure of the metamorphic layer under high-pressure cooling conditions, with the increase of flank wear width, the cutting force, cutting temperature, plastic strain, and strain rate showed a significant upward trend. Zhang et al. [30] found through experimental research on cutting GH4169 under high-pressure cooling that the degree of influence of cutting amount and coolant pressure on tool vibration was in order of feed speed, cutting depth, cooling pressure, and cutting speed. Liu et al. [31] used AdvantEdge finite element software to simulate the cutting of Inconel 718 under high-pressure cooling. The results showed that the greatest influence on the main cutting force was the cutting depth, and the greatest influence on the surface roughness was the feed rate. Wang et al. [32] found through simulation analysis of tool friction performance under dry cutting and high-pressure cooling conditions that the friction coefficient between tool and chip under high-pressure cooling was lower than that under dry cutting.

In summary, although many scholars have conducted extensive research on cutting superalloys under high-pressure cooling, they have mainly studied the effects of high-pressure cooling-assisted cutting on tool wear, tool vibration, modified layer and friction coefficient between tool and chip, etc., respectively, while there are few comprehensive analyses of the influence of high-pressure coolant pressure on the characteristics of the cutting layer, especially for the study of determining the optimal coolant pressure for cutting GH4169 under high-pressure cooling. However, this study is of great significance for improving the machinability of superalloy GH4169 and its machining accuracy. Therefore, this paper applied the single factor test method to carry out the experiments of turning superalloy GH4169 under high-pressure cooling, atmospheric pressure cooling, and dry cutting. The focus was on exploring the influence of different cooling conditions and coolant pressure on the cutting layer characteristics of superalloys cut by PCBN tools under high-pressure cooling. Firstly, the cutting force was measured and quantitatively analyzed. Secondly, the surface roughness was observed by a surface roughness meter. Thirdly, the residual stress on the surface of the workpiece was measured by the X-ray diffraction method. Then, the chip morphology was observed by a super-depth-of-field microscope. Finally, the change in tool life with time under different cooling pressures was recorded by a stopwatch. The above research results provide theoretical support for the optimization of the machining process of PCBN tool cutting the superalloy GH4169 under high-pressure cooling.

## 2. Experimental Design of a PCBN Tool Turning Superalloy under High-Pressure Cooling

In order to make the superalloy processing more efficient, this paper focuses on research on the cutting performance of PCBN cutting tools turning superalloy under high-pressure cooling, systematically analyzing the cutting force, surface roughness, surface residual stress, chip shape, and tool life affected by varying cooling pressure and coolant conditions. The specific test conditions and scheme are shown below.

### 2.1. Test Conditions

**Figure 1:**
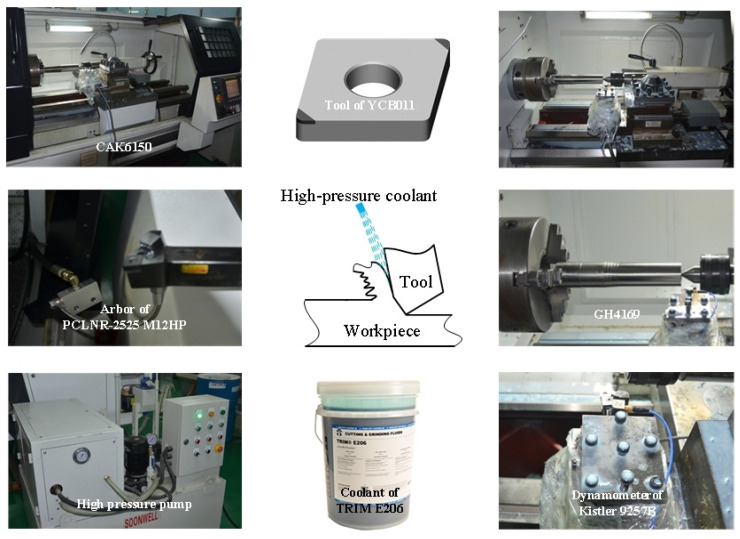
The experimental device.

The test was carried out with a CAK6150 lathe from Dalian Machine Tool Group, and the test device is shown in Figure 1. The workpiece has superalloy GH4169 rods with specifications of φ65 × 400 mm and φ100 × 380 mm. Its material properties are similar to those of the American and European Inconel 718 alloys. The specific chemical composition is shown in Table 1, and the physical and mechanical properties are shown in Table 2.

Using PCBN as the tool material, the cutting blade was an indexable turning blade manufactured by Zhuzhou Diamond Tools Company (Zhuzhou, China). The model and material brand of the blade were CNGA120408T series and YCB011, respectively. The tool holder was PCLNR-2525 M12HP, produced by Sandvik Company (Stockholm, Sweden), with a −6° rake angle, a 6° clearance angle, a 95° main bend angle, and a −6° cutting edge inclination angle.

The cutting was carried out by cylindrical turning, while the cooling was carried out by high-pressure cooling, atmospheric pressure cooling, and dry cutting. The coolant was TRIM E206, produced by Masda Chemical Company in the United States, with the high-pressure coolant and the atmospheric pressure coolant supplied by the external high-pressure pump and machine tool, respectively. In high-pressure cooling cutting experiments, the angle and distance of coolant injection were maintained constant.

Detection method: a TR200 handheld surface roughness meter was used to measure surface roughness; cutting force was measured by a Kistler 9257B, Winterthur, Switzerland; surface residual stress was measured by an IXRD X-ray stress meter; and tool life was measured by a stopwatch.

### 2.2. Test Scheme

In order to explore the influence of different cooling conditions and high-pressure coolant pressure on the cutting layer characteristics of PCBN tool cutting GH4169, in this paper the single factor test method was applied, that is, during the test process, the geometric parameters and cutting parameters of the tool were kept unchanged, and only the cooling conditions were changed. The setting of the specific test scheme is shown in Table 3.

## 3. Experimental Results and Discussion of PCBN Tool Turning Superalloy under High-Pressure Cooling

### 3.1. Research on the Cutting Force of Cutting GH4169 under High-Pressure Cooling

**Figure 2:**
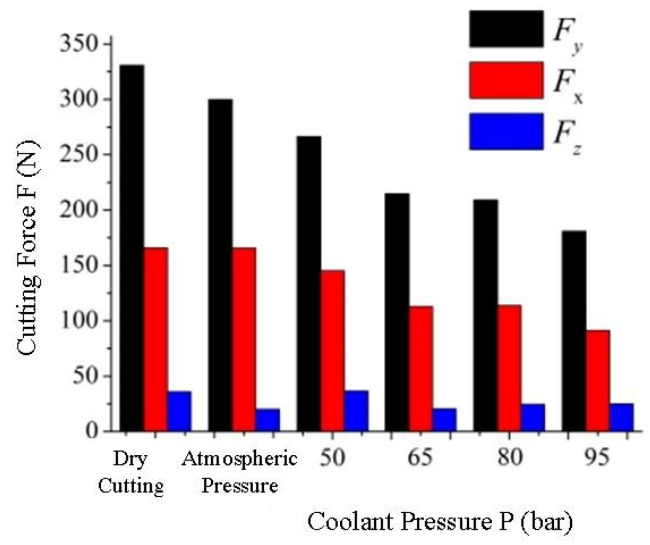
Effect of cooling conditions on cutting forces (v_c_ = 160 m/min, f = 0.1 mm/r, a_p_ = 1 mm).

Based on the single factor test method, the effect curves of varying cooling pressure and coolant conditions on cutting force were obtained, as shown in Figure 2, where F_x_ represents axial force, F_y_ represents radial force, and F_z_ represents tangential force. As can be seen from Figure 2, the cutting force is greatly affected by varying cooling pressure and coolant conditions. Compared with atmospheric pressure cooling and dry cutting, the cutting force of cutting GH4169 under high-pressure cooling is obviously lower. In addition, as the coolant pressure increases, the cutting force decreases. Through data analysis of the obtained cutting forces, it is found that when cutting GH4169 under high-pressure cooling compared to atmospheric pressure cooling, the axial force F_x_ decreases by 12% to 45%, the radial force F_y_ decreases by 11% to 39%, and the tangential force F_z_ remains essentially unchanged. Compared with dry cutting, the axial force F_x_ decreases by 12% to 45%, the radial force F_y_ decreases by 19% to 45%, and the tangential force F_z_ remains essentially unchanged.

The lubrication characteristics of the coolant can change the friction state between the tool and the chip, and the coolant under higher pressure is conducive to its penetration into the contact area of the tool–chip, forming boundary lubrication, thereby reducing the friction coefficient between the tool and the chip and reducing the cutting force. The cooling characteristics of the coolant can reduce the temperature of the cutting area, and under the action of high pressure, the coolant can further improve the heat transfer coefficient of the cutting area, reduce the cutting temperature, ensure tool strength, and keep the tool edge intact. Therefore, compared to dry cutting, superalloy cutting with atmospheric and high-pressure cooling has a lower cutting force. As the coolant pressure increases, the chips are prone to breaking, and the friction between the tools and chips further decreases. Therefore, the cutting force gradually decreases as the coolant pressure increases. It can also be seen from Figure 2 that the high-pressure coolant pressure has a great influence on the axial force F_x_ and radial force F_y_ but a small influence on the tangential force F_z_.

### 3.2. Research on the Machined Surface Roughness of Cutting GH4169 under High-Pressure Cooling

**Figure 3:**
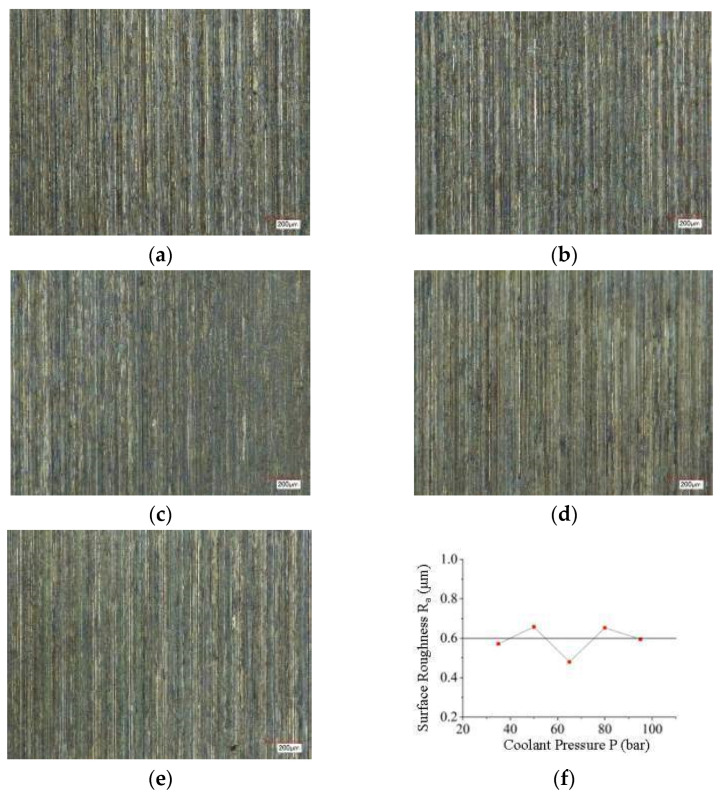
Effect of different coolant pressures on machined surface topography and surface roughness under high-pressure cooling (X200) (v_c_ = 125 m/min, f = 0.05 mm/r, a_p_ = 0.4 mm); (**a**) *p* = 35 bar (Ra = 0.576 μm); (**b**) *p* = 50 bar (Ra = 0.658 μm); (**c**) *p* = 65 bar (Ra = 0.480 μm); (**d**) *p* = 80 bar (Ra = 0.653 μm); (**e**) *p* = 95 bar (Ra = 0.595 μm); (**f**) Curve of surface roughness versus coolant pressure *p*.

Based on the experimental study of PCBN tool cutting GH4169 under high-pressure cooling, the regular curve of machined surface roughness varying with coolant pressure was obtained. As can be seen from Figure 3, there is no obvious linear relationship between GH4169’s machined surface roughness and the high-pressure coolant pressure, that is, the high-pressure coolant pressure has little effect on the surface roughness.

This is mainly because during the cutting deformation process of the workpiece, the machined surface quality is mainly related to the third deformation zone, while the high-pressure coolant injection method of the test mainly acts on the second deformation zone, that is, the rake face of the tool, so the surface roughness is less affected by the high-pressure coolant pressure. In addition, the chip is easily broken under the impact of high-pressure coolant. During the breaking process, the cutting force fluctuates, causing cutting vibration to act on the tool, causing the tool to vibrate, and causing intermittent contact between the PCBN tool and the surface of the workpiece to be machined. This ultimately results in intermittent changes in the residual height of the surface of the workpiece to be machined, causing irregular peaks and valleys to form on the surface of the workpiece and fluctuations in the surface roughness of the workpiece to be machined, as shown in Figure 3f. When the workpiece is processed under high-pressure cooling, the cutting temperature is lower, and the surface roughness is not sensitive to changes in cutting temperature.

### 3.3. Research on Machined Surface Residual Stress While Cutting GH4169 in High-Pressure Cooling

**Figure 4:**
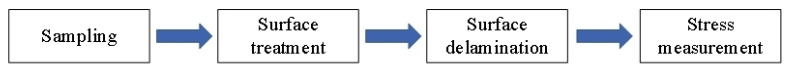
The measurement process for residual stress.

In the current non-destructive testing techniques for residual stress on machined surfaces, X-ray diffraction has high accuracy, strong reliability, and simple operation. It is a commonly used method for non-destructive testing [33]. X-ray diffraction can directly measure the residual stress distribution on the workpiece surface. However, if the residual stress is to be measured at a certain depth from the workpiece surface, it is necessary to take the corrosion stripping method of the workpiece, expose the surface layer, and then measure the residual stress of the layer. The general measurement process to measure the surface residual stress distribution along the depth of the layer is shown in Figure 4.

**Figure 5:**
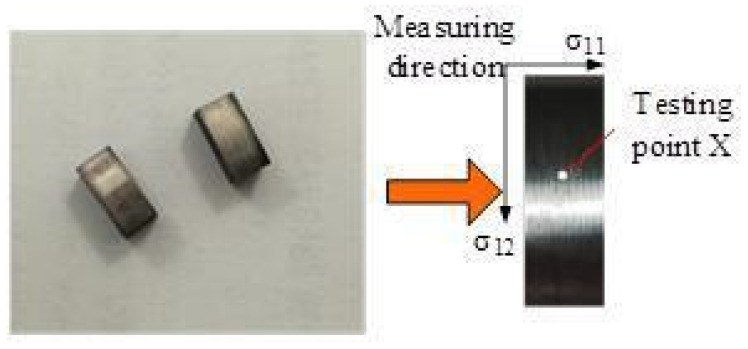
Test of the sample.

**Figure 6:**
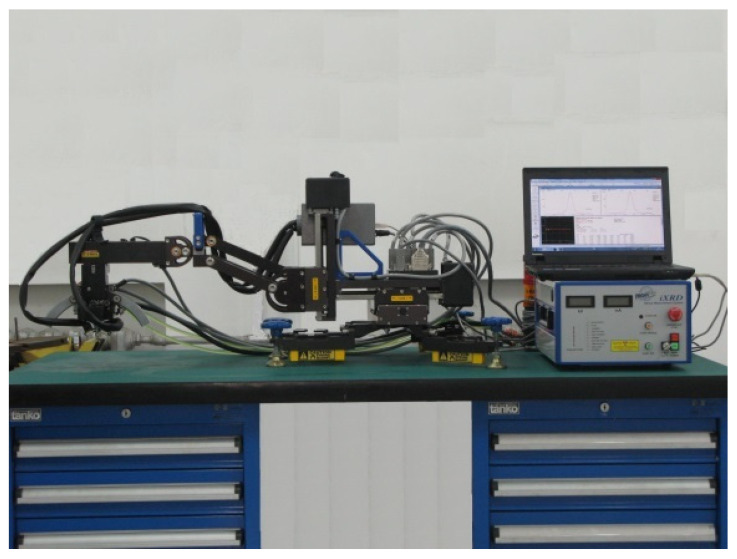
X-ray stress meter IXRD.

The workpiece left over from the high-pressure cooling cutting test was made into a 10 × 5 × 5 size sample by wire cutting, which was used to detect residual stress on the surface under various cooling conditions. The sample is shown in Figure 5. The X point in the figure is the point to be measured for surface residual stress. In addition, surface residual stress at the X point is measured in the feed and cutting speed directions, respectively. The residual stress was tested with an X-ray IXRD stress meter, as shown in Figure 6. The test parameters were set as Mn target material, voltage of 20 kV, current of 4 mA, quasi-visual size of 2 mm, exposure time of 5 s, and exposure times of 10. The X-ray diffraction peak was analyzed by the X-ray diffraction method, and the parameters were fitted. Finally, the residual stress of the machined surface of GH4169 was measured.

**Figure 7:**
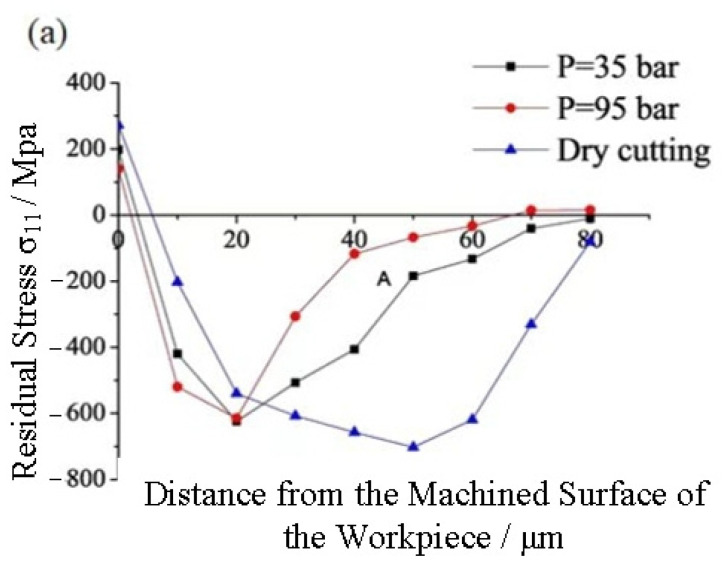

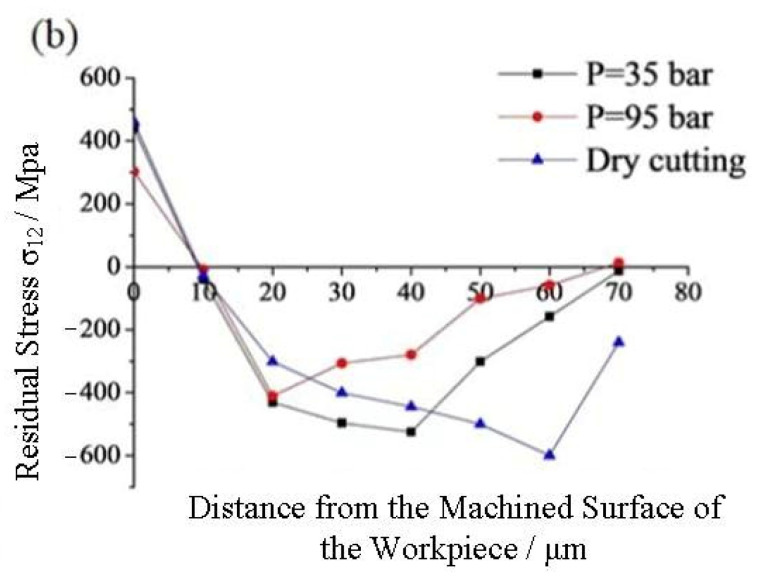
Influence of various cooling conditions on the residual stress of a machined surface. (v_c_ = 125 m/min, f = 0.05 mm/r, a_p_ = 0.4 mm); (**a**) Residual stress in the feed direction, (**b**) residual stress in the direction of cutting speed.

An X-ray IXRD stress meter was used to determine the variation law of residual stress on GH4169’s surface under various cooling conditions, as shown in Figure 7. As can be seen from Figure 7, no matter whether the workpiece is cut under dry cutting or high-pressure cooling conditions, the surface of the workpiece presents residual tensile stress, while its interior presents residual compressive stress. At the same time, the maximum value of the absolute values of the surface residual tensile stress and the internal residual compressive stress of the workpiece under the cutting condition of the former is greater than that of the latter, and the maximum value is further reduced when the coolant pressure becomes larger. The combined effects of mechanical force and heat are primarily responsible for the formation of residual stress on the surface being machined. When cutting a superalloy under high-pressure cooling, the coolant easily enters the tool–chip contact area under high pressure, which is beneficial to form boundary lubrication in this area and reduce friction between them, so that the cutting force is reduced. Moreover, the high-pressure coolant can increase the heat transfer coefficient in the cutting area and reduce the cutting temperature. The impact, cooling, and lubrication characteristics of high-pressure coolant reduce the cutting force and cutting heat in the superalloy cutting process, while the cutting force and cutting heat are the main factors affecting the residual stress of the surface. Therefore, cutting GH4169 under high-pressure cooling can reduce the surface residual stress of the workpiece.

### 3.4. Research on the Chip Morphology of GH4169 Cutting under High-Pressure Cooling

#### 3.4.1. Research on the Macroscopic Morphology of the GH4169 Chip

**Figure 8:**
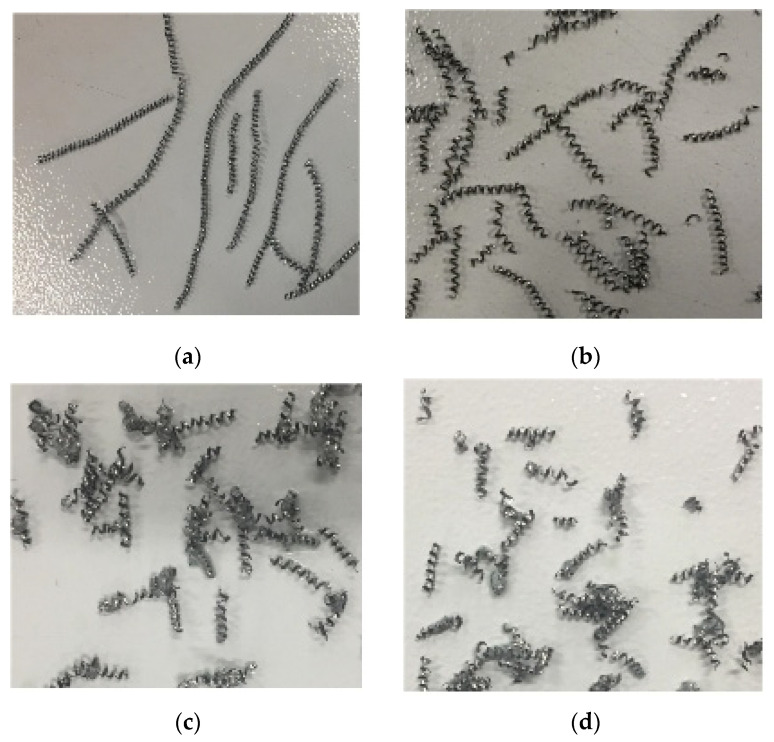

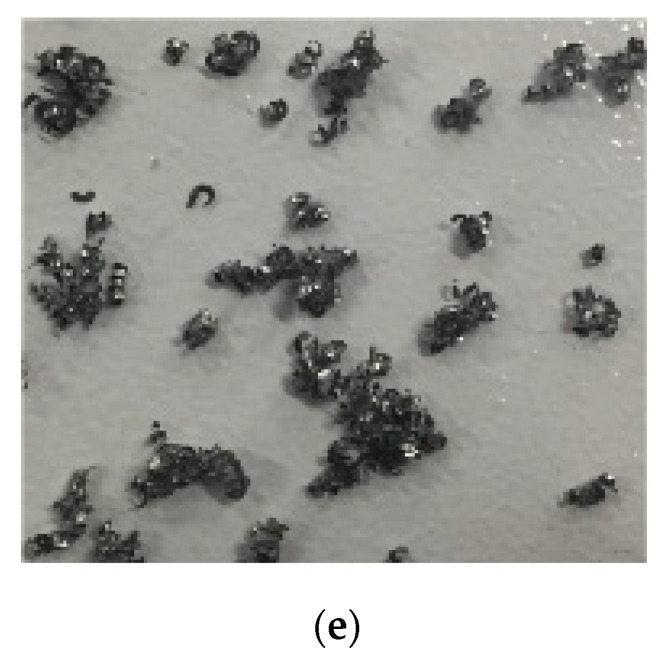
The influence of cooling pressure on the macro-shape of the chip (v_c_ = 125 m/min, f = 0.05 mm/r, a_p_ = 0.4 mm); (**a**) *p* = 35 bar; (**b**) *p* = 50 bar; (**c**) *p* = 65 bar; (**d**) *p* = 80 bar; (**e**) *p* = 95 bar.

In this paper, the single factor test method was used to conduct experimental research on cutting GH4169 with a PCBN tool under high-pressure cooling, and the effect law of high-pressure coolant pressure on the macroscopic shape of the chip was obtained, as shown in Figure 8. As can be seen from Figure 8, when cutting superalloys under high-pressure cooling with the same cutting amount, as the coolant pressure increases the chips gradually change from long spiral chips to short chip chips, and the chip length becomes smaller. Moreover, when the coolant pressure is 95 bar, the chips show a C-shaped shape.

From the above test results, it can be concluded that the chip breaking ability gradually increases with the increase of coolant pressure, and the higher the coolant pressure, the easier the chip breaking. This shows that high-pressure cooling cutting is effective in solving the problem where the superalloy chip is difficult to break.

#### 3.4.2. Research on the Micromorphology of the GH4169 Chip

**Figure 9:**
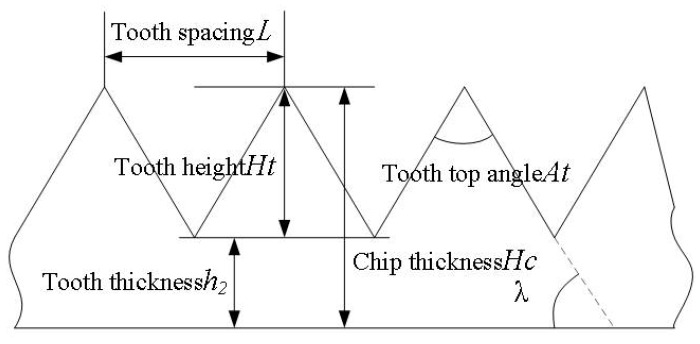
The characterization of the degree of chip serration.

The typical machining characteristic of a superalloy is the sawtooth shape of its chips. The geometric characterization of serrated chips is shown in Figure 9. Compared with strip continuous chips, serrated chips have the geometric characteristics of periodicity and uneven thickness. In Figure 9, L is the tooth spacing; h_2_ is the tooth thickness; H_t_ is the tooth height; H_c_ is the chip thickness; A_t_ is the addendum angle; λ_0_ is the chip shear angle. The formation of serrated chips makes the cutting force fluctuate in the process of superalloy cutting, which affects the tool life and then affects the accuracy of the workpiece surface and machining efficiency. Therefore, the morphology of GH4169 serrated chips in high-pressure cooling was analyzed in this paper, and the influence law of high-pressure coolant on the formation of serrated chips was obtained through a high-pressure cooling test. According to Schulz’s mathematical model, the degree of serrated chip serration and its geometric characteristics were quantitatively analyzed [34], as shown in Equation (1). At the same time, the serrated frequency [35] was obtained by using the mathematical model proposed by M.C. Shaw, as shown in Equation (2), to accurately characterize the superalloy serrated chip.

(1) $$G_{s}=\frac{H_{c}-h_{2}}{H_{c}}$$

(2) $$f_{chip}=\frac{v_{chip}}{L}$$

In the formula, v_chip_ can be obtained from Equation (3).

(3) $$v_{chip}=\frac{v_{c}fsink_{r}}{a_{ch}^{\prime }}$$

In the formula, v_c_ is the cutting speed; f is the feed; k_r_ is the main deflection angle; and a^′^_ch_ is the average chip thickness.

Serrated chips produced by PCBN tool cutting GH4169 under high-pressure cooling were collected, then ground and polished to obtain the chip metallographic sample. Subsequently, a VHX-1000 ultra-deep field microscope was used to analyze the serrated chips.

**Figure 10:**
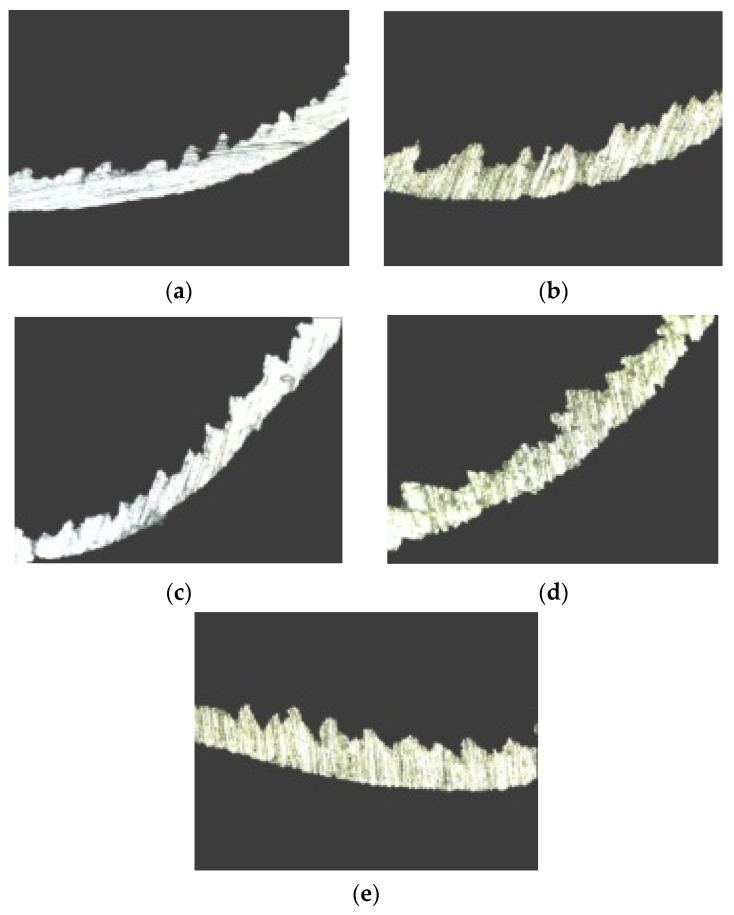
Effects of coolant pressure on the morphology of serrated chips; (**a**) *p* = 35 bar; (**b**) *p* = 50 bar; (**c**) *p* = 65 bar; (**d**) *p* = 80 bar; (**e**) *p* = 95 bar.

**Figure 11:**
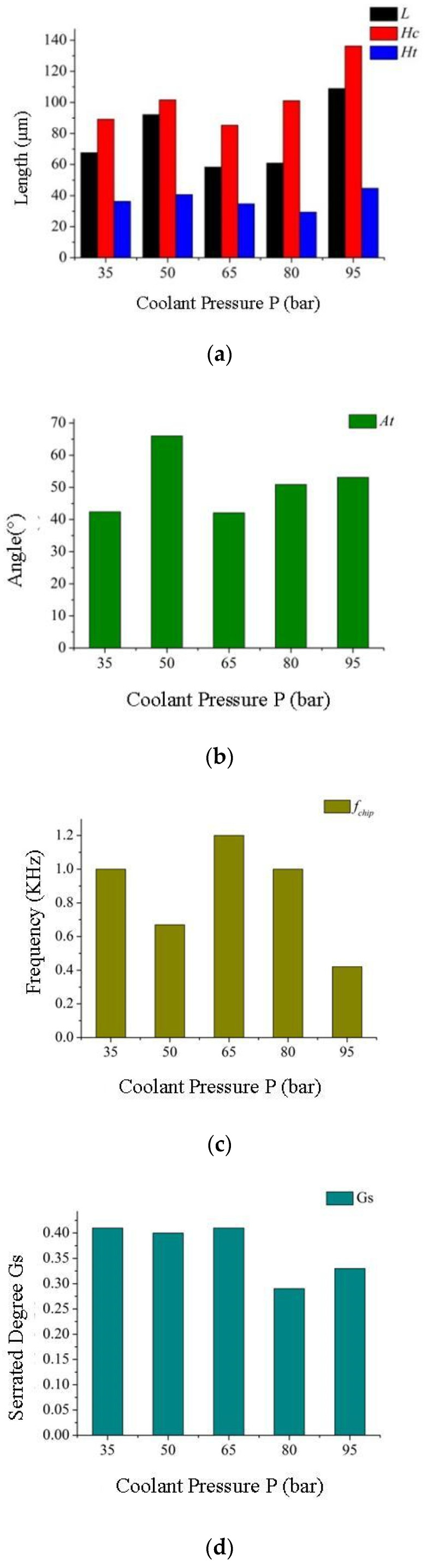
Effect of coolant pressure on the microcosmic characteristics of serrated chips (v_c_ = 125 m/min, f = 0.05 mm/r, a_p_ = 0.4 mm); (**a**) The influence of coolant pressure on serrated chip tooth spacing, tooth height, and chip thickness; (**b**) The influence of coolant pressure on serrated chip addendum angle; (**c**) The effect of coolant pressure on the serration generation frequency of serrated chips; (**d**) The effect of coolant pressure on the serrated degree of serrated chips.

Figure 10a–e show changes in serrated chip morphology affected by high-pressure coolant pressure when the coolant pressure is 35 bar, 50 bar, 65 bar, 80 bar, and 95 bar, respectively. Figure 11a–d show changes in the chips’ serrated geometric characteristics affected by high-pressure coolant pressure.

As can be seen from Figure 10, the high-pressure coolant influences, to some extent, how serrated chips are formed.

As can be seen from Figure 11, with the same cutting amount, the length between adjacent serrations, chip thickness, and addendum height tend to increase as the high-pressure coolant pressure increases, while the frequency and degree of chip serration tend to decrease. The main reason for this is that the generation of cutting heat plays a dominant role in the formation of serrated chips, and high-pressure coolant sprayed into the cutting area can effectively reduce the heat generated during the machining process. The temperature of the shear area will decrease with the increase of the cooling hydraulic pressure, weakening the softening effect of the material and slowing down the rate of instability of the shear surface material due to adiabatic shearing, that is, the angle between the inside of the serrated segment is increased and the adiabatic shear angle is reduced, resulting in an increasing trend in chip thickness. The decrease in cutting temperature inhibits the adiabatic shear phenomenon of chips in the shearing area and thus reduces the degree of chip serration. In addition, the bending moment on the chips will increase with the increase of cooling hydraulic pressure, leading to an increase in chip deformation, which reduces the contact length between the serrations and also reduces the degree of chip serration.

### 3.5. Research on PCBN Tool Life under High-Pressure Cooling

In this paper, based on the single factor test method, the variation law in tool life of the PCBN-cut superalloy GH4169 in the presence of high-pressure cooling was analyzed. During the cutting test, when the VB value of the PCBN tool flank surface is greater than 300 μm, the tool is considered to meet the blunting standard, the test is stopped and then the cutting time is recorded.

**Figure 12:**
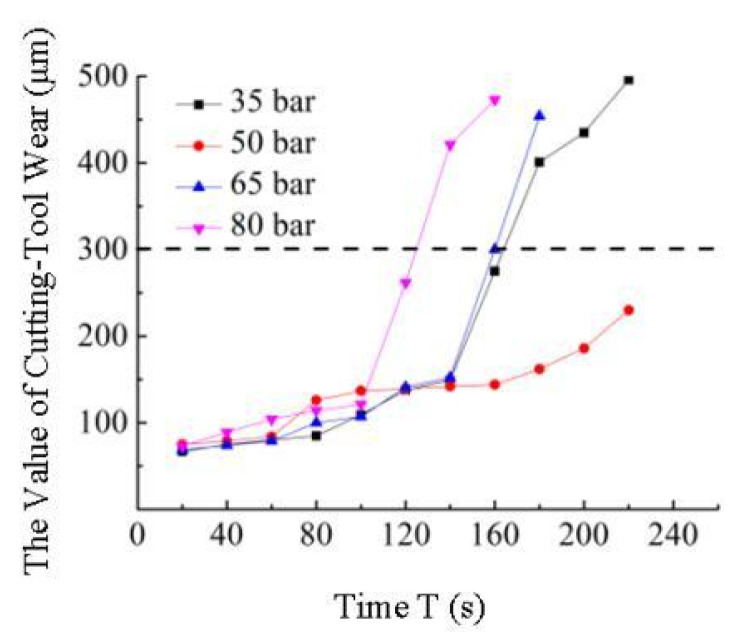
Effect of different coolant pressures on tool life under high-pressure cooling (v_c_ = 125 m/min, f = 0.05 mm/r, a_p_ = 0.4 mm).

The variation law of PCBN tool life under different coolant pressures was obtained by the single factor test, as shown in Figure 12. With the cutting amount unchanged, it can be seen from Figure 12 that when the cutting time is less than 100 s, PCBN tool wear is relatively stable, and the VB value does not differ significantly, indicating that the tool edge shape remains intact at the initial cutting stage. When the cutting time exceeds 100 s, the VB value of the PCBN tool flank changes greatly. In particular, the wear degree of the PCBN tool is significantly increased when the coolant pressure is 80 bar, and it quickly reaches the blunt standard. When the coolant pressure is 35 bar, 50 bar, or 65 bar, the tool wear is relatively stable, but when the cutting time exceeds 140 s, the tool VB value also increases sharply, indicating that the tool enters the later stage of severe wear. When the coolant pressure is 50 bar, PCBN tool wear is relatively stable, and there is no sharp increase in VB value. This indicates that when the PCBN tool is cutting GH4169 under high-pressure cooling, in order to ensure tool life, the coolant pressure should not be too large, and it is more appropriate at 50 bar.

## 4. Conclusions

In this paper, experimental research on the PCBN tool cutting superalloys under high-pressure cooling was carried out. The variation law of cutting force, surface roughness, surface residual stress, chip shape, and tool life under different cooling conditions was emphatically analyzed. The results are as follows:

(1) The axial force F_x_ and radial force F_y_ produced by PCBN tool cutting GH4169 under high-pressure cooling conditions are greatly influenced by coolant pressure, but the tangential force F_z_ is not significantly affected. Within the range of selected high-pressure cooling test parameters, the radial force can be reduced by 11~39% and 19~45%, respectively, compared to atmospheric cooling cutting and dry cutting.
(2) When cutting GH4169 with the PCBN tool in the presence of high-pressure cooling, the high-pressure coolant has little effect on the roughness of the machined surface but has a great effect on the residual stress of the machined surface. The absolute value of the maximum residual stress on the machined surface of GH4169 under high-pressure cooling is lower than that under dry cutting conditions, and the maximum value is further reduced when the coolant pressure becomes larger.
(3) According to the macroscopic morphology of chips, the high-pressure coolant has a significant effect on improving chip breaking ability. As the coolant pressure reaches 95 bar, the chip breaks quickly and presents C-type chips. According to the microscopic morphology of chips, the length between adjacent serrations, chip thickness, and addendum height tend to increase as the high-pressure coolant pressure increases.
(4) To ensure the service life of the PCBN tool while cutting GH4169 under high-pressure cooling, the coolant pressure should not be too large, and 50 bar is more appropriate.

The above research results can optimize the appropriate coolant pressure for PCBN tool cutting superalloy under high-pressure cooling and provide a theoretical basis and technical support for the promotion of high-pressure cooling cutting technology and superalloy processing with high efficiency and quality. Future research directions can optimize the coolant pressure through the analysis of the coupling characteristics of cutting force and cutting temperature in high-pressure cooling cutting superalloys.

## Figures and Tables

**Table 1:** Chemical composition of GH4169 (%).

Ni	Cr	Mo	Nb	Ti	Al	C	Si	Mn	Fe
53.38	18.40	3.0	5.13	1.1	0.38	0.017	0.062	0.05	residual

**Table 2:** Physical and mechanical properties of GH4169 (20 °C).

Density ρ/(g/cm^3^)	Yield Strength δ0.2/MPa	Tensile Strength δb/MPa	Elongation δS/%	Shrinkage Ratio ψ/%	Impact Ductility ak/(MJ/m^2^)	Hardness HB
8.28	1152	1376	19	45.5	348	410–420

**Table 3:** Test scheme.

Coolant P/bar	Cutting Amount	Chamfer Angle	Chamfer Width
dry cutting, atmospheric pressure, 35, 50, 65, 80, 95	v_c_ = 125 m/min, 160 m/min, f = 0.05 mm/r, 0.1 mm/r, a_p_ = 0.4 mm, 1 mm	15°	0.15 mm
